# Prognostic Value of Basal Serum Thyroglobulin Levels, but Not Basal Antithyroglobulin Antibody (TgAb) Levels, in Patients with Differentiated Thyroid Cancer

**DOI:** 10.4274/mirt.39200

**Published:** 2014-06-05

**Authors:** Isa Neshandar Asli, Ali Shafiepour Siahkali, Babak Shafie, Hamid Javadi, Majid Assadi

**Affiliations:** 1 Taleghani Hospital, Shahid Beheshti University of Medical Sciences, Department of Nuclear Medicine, Tehran, Iran; 2 Golestan University of Medical Sciences (GUOMS), Golestan Research Center of Gastroenterology and Hepatology (GRCGH), Gorgan, Iran; 3 Bushehr University of Medical Sciences, The Persian Gulf Nuclear Medicine Research Center, Bushehr, Iran

**Keywords:** thyroid cancer, thyroglobulin, anti-thyroglobulin

## Abstract

**Objective:** The prognostic values of serum thyroglobulin (Tg) and antithyroglobulin antibody (TgAb) levels, measured immediately before I-131remnant ablation in patients with differentiated thyroid cancer (DTC), have been advocated by some researchers; however, it had controversial outcomes. This study was carried out to examine this dilemma and to check the clinical significance of basal serum Tg and TgAb levels and postablation iodine 131whole body scan(WBS) findings in DTC patients.

**Methods:** In this retrospective study, the records of 500 patients with differentiated thyroid cancer, who had undergone treatment between 2003 and 2010, were assessed. Of those, 149 patients with results of basal serum thyroglobulin concentration and whole body scan using radioactive iodine were included. Age, sex, tumour histology, basal thyroglobulin (Tg), anti-thyroglobulin (TgAb) and TSH concentration, radioactive iodine doses in each hospitalization, numbers of hospitalization, and results of whole body scans were recorded. The relationship among basal Tg, TgAb, TSH, and whole body scan with hospitalization number and total radioactive iodine doses were assessed.

**Results:** A total of 149 patients, including 123 (83%) females and 26 (17%) males, with a mean age of 40±15 years, took part in the study. The mean (SD) basal Tg, TgAb, and TSH were 91.7±169.2 ng/mL (0.1-1000 ng/mL), 250±893 U/mL (0-9000 U/m L), and 64.8±61.5 µU/mL (30-689 U/mLµ), respectively. A total of 52 (34.9%) cases had TgAb levels greater than 100 U/mL. The mean basal Tg in patients who were admitted three or more times was significantly greater than that of patients with one hospitalization (p=0.026). In addition, the mean of Tg in patients who received 7.4 GBq radioactive iodine or less was significantly lower than the others (p=0.003). The mean of TgAb and TSH were not different between these groups. In the results of the whole body scans, patients with metastasis had higher frequency of hospitalization (p=0.010) and received higher radioactive iodine levels (p<0.001).

**Conclusions:** The findings of this study showed that, in differentiated thyroid cancer, lower basal serum Tg levels and absence of metastasis in radioiodine scan after ablation treatment were correlated with fewer hospitalizations and lower doses of radioactive iodine. Basal TgAb and TSH had no relation. Therefore, it seems that basal Tg could help us in determining which patients need aggressive treatment.

## Abstract (tr)

**Amaç:** Diferansiye tiroid kanserli (DTK) hastalarda I-131 ile rezidü ablasyonundan hemen önce ölçülen serum tiroglobulin (Tg) ve antitiroglobulin antikoru (TgAb) düzeylerinin prognostik değeri bazı araştırıcılar tarafından ileri sürülmekle beraber, tartışmalı sonuçlar mevcuttur. Bu çalışma, DTK’li hastalarda bu ikilemi incelemek ve bazal serum Tg ve TgAb düzeylerinin ve ablasyon sonrası I-131 tüm vücut tarama bulgularının klinik önemini araştırmak için planlandı.

**Yöntem:** Bu retrospektif çalışmada 2003 ve 2010 yılları arasında tedavi alan DTK’lı 500 hastanın kayıtları incelendi. Bu hastalar arasından bazal serum Tg konsantrasyonu ve radyoaktif iyot ile tüm vücut tarama sonuçları olan 149 hasta dahil edildi. Yaş, cinsiyet, tümör histolojisi, bazal Tg, TGAb ve TSH konsantrasyonları, her yatıştaki radyoaktif iyot dozu, yatış sayısı ve tüm vücut tarama sonuçları kaydedildi. Bazal Tg, TGAb, TSH ve tüm vücut tarama ile yatış sayısı ve toplam radyoaktif iyot dozu arasındaki ilişki araştırıldı.

**Bulgular:** Yaş ortalaması 40±15 olan, 123 (%83) kadın ve 26 (%17) erkek olmak üzere toplam 149 hasta çalışmaya alındı. Ortalama (SD) bazal Tg, TgAb ve TSH değerleri, sırasıyla 91,7±169,2 ng/mL (0,1–1000 ng/mL), 250±893 U/mL (0-9000 U/m L) ve 64,8±61,5 µU/mL (30-689 U/mLµ) idi. Toplam 52 (%34,9) hastanın TgAb düzeyleri 100 U/mL den yüksekti. Üç ya da daha fazla sayıda yatış yapılan hastaların ortalama bazal Tg düzeyleri, tek yatış yapılan hastalardan anlamlı olarak yüksekti (p=0,026). Ayrıca, 7,4 GBq ya da daha az radyoiyot alan hastaların ortalama Tg değerleri diğerlerine göre anlamlı olarak daha düşüktü (p=0,003). Ortalama TgAb ve TSH gruplar arasında farklılık göstermiyordu. Tüm vücut tarama sonuçlarına göre, metastazlı hastalarını hastaneye yatış frekansları daha yüksekti ve daha yüksek radyoaktif iyot alımı mevcuttu (p<0,001).

**Sonuç:** Bu çalışmanın bulguları, diferansiye tiroid kanserinde, düşük bazal serum Tg düzeyleri ve ablasyon sonrası radyoiyot taramada metastaz olmamasının daha az hastaneye yatış sayısı ve daha düşük radyoaktif iyot dozları ile korele olduğunu gösterdi. Bazal TgAb ve TSH korelasyon göstermedi. Bu nedenle, bazal Tg hangi hastaların agresif tedaviye gereksinim duyduğunu belirlemede yardımcı olabileceği düşünüldü.

## INTRODUCTION

Initial treatment of differentiated thyroid carcinoma (DTC) consists of total or near total thyroidectomy, together with radioiodine ablation (1). Throughout follow-up examinations, an excellent association between the presence of normal and/or malignant thyroid tissue and serum stimulated thyroglobulin (Tg) levels has been noted (2). In this query, some studies have proposed the possibility of using high Tg values, determined just before 131I remnant ablation (basal Tg), as a prognostic indicator (3,4). 

On the other hand, it is critically interfered in the presence of antithyroglobulin antibody (TgAb) (5). The percentage of TgAb in DTC patients have been reported in the 10%-30% range (6,7). Some controversy exists regarding the clinical significance of TgAb in DTC patients. Some investigations have presented higher frequencies of recurrent or persistent disease related to persistent TgAb (5,8,9,10), but some studies have not demonstrated such correlations (11,12,13). The later prospective investigations have reported that TgAb levels did not affect disease aggravation, and that TgAb diminished gradually after surgery in most cases throughout a three-year follow-up (13,14). 

However, no complete statement exists on the clinical significance of elevated basal serum Tg and basal TgAb levels in DTC patients. 

We investigated the clinical significance of serum Tg, TgAb, and TSH levels, measured immediately before I-131 remnant ablation in patients with differentiated thyroid carcinoma. We also assessed the clinical effect of postablation 131 iodine whole body scan (WBS) findings. By means of this data, we may be able to evaluate the above tests as early prognostic markers for predicting disease outcome. 

## MATERIALS AND METHODS

**Participants and Study Design**

This retrospective study recruited 500 patients who had a history of DTC over a period of eight years, from 2003 to 2010. Patients were followed for surgical and pathologic findings, status of DTC at the time of the initial surgery, extent of metastasis at the time of radioisotope scanning, subsequent operations, clinical findings, and serum Tg, TgAb, and TSH levels. We excluded patients with insufficient data; as such, 149 patients fulfilled the eligibility criteria, provided informed consent, and participated in the study. Patients with abnormal findings on follow-up whole-body scintigraphy with I-131 (WBS) and/or elevated Tg levels were considered for repeated therapy. WBS and Tg levels were measured after stopping of levothyroxine for a month and two weeks of liothyronine. Serial monitoring was carried out using serum Tg and TgAb measurements and WBS. At the time of the diagnostic WBS, all patients had serum TSH levels above 30 mIU/l. The post-therapy scans were performed just before the patient was released after I-131 therapy. This study complies with the Declaration of Helsinki, and it was approved by the institutional ethics committee of our University. 

**Acquisition Protocols**

For 1-131 scintigraphy, the patients orally received 185 MBq (5 mCi) I-131, and scintigraphy was performed 48 h later. Planar and single photon emission computed tomography (SPECT) images were obtained by means of a double-head gamma camera (ADAC Genesys, Milpitas, CA, USA) with a high-energy, parallel hole collimator, where the energy setting was at 364 keV ± 10%. The images were observed and interpreted by two experienced nuclear medicine physicians. 

**Measurement of Serum Thyroglobulin**

Serum thyroglobulin and anti-Tg antibody levels were determined by radioimmunoassay, using commercial kits (BRAHMS Tg-plus DYNO test and BRAHMS anti-Tgn DYNO test). In addition, serum TSH was measured with a third-generation double antibody assay. 

**Statistical Methods**

Continuous variables are expressed as mean ± SD, and categorical variables as the absolute values and percentages. The relationship among basal Tg, TgAb, and TSH levels and the number of hospitalizations and total radioactive iodine dose were determined by one-way analysis of variance (ANOVA) and post hoc Bonferroni tests. In addition, a Chi-square test was used to assess the relation of the results of post-ablative I-131 WBS with number of hospitalizations and total radioactive iodine dose. In addition, a Chi-square test was used to compare differences between categorical variables. P-values of 0.05 or less were considered to be significant. Statistical analysis was performed utilizing SPSS (SPSS Inc., Chicago, IL, USA), version 18. 

## RESULTS

**Patient Characteristics**

A total of 149 patients, including 123 (83%) females and 26 (17%) males with a mean age of 40 ± 15 years took part in the study. A total of 138 (93%) patients had the papillary type of differentiated thyroid cancer (PCDTC), and 11 (7%) cases had the follicular type of differentiated thyroid cancer (FCDTC). 

In pathology, 79 patients (59%) had no extension of disease beyond the thyroid, 11 (7%) had local extension (capsular or vascular), 44 (30%) had lymph node involvement with or without local extension, and 15 (10%) had distant metastasis. 

The mean and SD of basal Tg, TgAb, and TSH were 91.7±169.2 ng/mL (0.1-1000 ng/mL), 250±893 U/mL (0-9000 U/m L) and 64.8±61.5 µU/mL (30-689 U/mLµ), respectively. A total of 52 (34.9%) cases had TgAb levels greater than 100 U/mL. 

There is higher basal Tg in the FCDTC relative to PCDTC type (261.10±234.16 vs. 76.93±156.30 ng/mL; p value=0.00), while there is no significant difference in the basal levels of TgAb (53.38±44.74 vs. 269.02±934.83 U/mL; p value=0.44) between two types. TSH levels were higher in PCDTC rather than FCDTC (68.40±62.89 vs 29.50±19.28 U/mLµ; p value=0.04). 

Likewise, there were higher basal Tg levels in the patients with metastasis than in the patients without metastasis (216.61±235.03 vs. 69.67±148.64 ng/mL; p value=0.02, while there were no differences in TgAb (71.83±146.57 vs. 261.27±941.34; p value=0.37) or TSH levels (216.61±235.03 vs. 69.67±148.64; p value=0.52). 

At the initial hospitalization, 65 (44%) patients received 3.7 GBq radioiodine, 66 (44%) patients received 5.55 GBq, and 18 patients received more than 5.55 GBq radioiodine. 

In the post-treatment WBS, 118 (79%) patients showed increased activity in the thyroid bed, 48 (32%) in the cervical lymph node, 30 (20%) in the mediastinal lymph node, 14 (9%) in the lungs, 9 (6%) in the bone, and 2 (1%) in the liver. Distant metastases were seen in 21 (14%) cases. 

The patients were also categorized according to their post-treatment WBS findings. In total, 91 (61%) patients had negative or only thyroid bed activity, 37 (25%) had lymph node involvement, with or without thyroid bed, and 21 (14%) cases had metastasis, with or without thyroid bed or lymph node involvement, on WBS. The number of hospitalizations was as follows: one in 62 (42%) patients, two in 51 (34%) patients, and three or more in 36 (24%) patients. 

The cumulative I-131 dose was as follow: 75 (50%) patients received 7.4 GBq or less, 57 (38%) patients received 7.437-18.5 GBq, and 17 (11%) received more than 18.5 GBq. 

**Figure 1:**
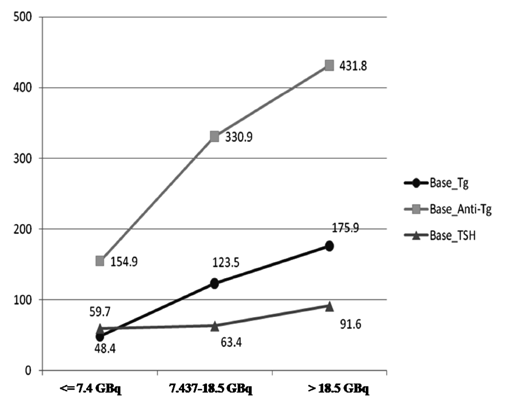
The mean basal Tg, TgAb, and TSH levels in three different groups, according to cumulative doses of 131I.

When comparing basal Tg, TgAb, and TSH levels in three different groups, according to cumulative doses of I-131, using an ANOVA test, only the mean of basal Tg in patients who received 7.4 GBq or lower cumulative doses of I-131 was significantly lower than the others (p=0.003). A post hoc Bonferroni test demonstrated that this difference was between the patients with 4 GBq or less and the other two groups. The means of TgAb and TSH were not different between these groups (p-value>0.05) (Table 1, Figure 1). 

**Figure 2:**
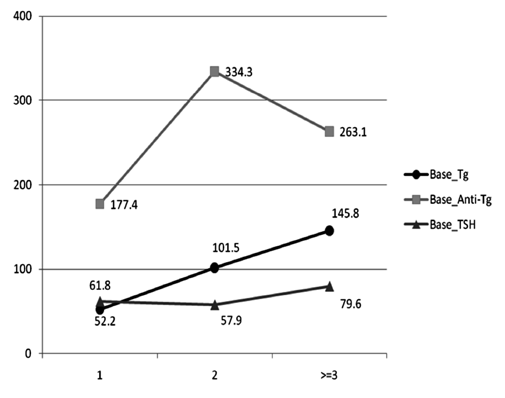
The mean basal Tg, TgAb, and TSH levels in three different groups, according to number of hospitalizations.

In addition, the mean of basal Tg in patients who were admitted three or more times was significantly more than that of patients with one hospitalization (p=0.026). The mean of TgAb and TSH were not different between these groups (Table 2, Figure 2). 

Furthermore, we compared the cumulative radioiodine dose and hospitalization number variables among the three groups according to the findings of post-treatment whole-body scans, using a chi-square test. In the results of post-treatment whole-body scan, patients with metastasis had a higher number of hospitalizations (p=0.010) and received higher levels of radioactive iodine (p<0.001). 

## DISCUSSION

The findings of this study showed that, in differentiated thyroid cancer, lower basal serum Tg levels and the absence of metastasis in radioiodine scan after ablation treatment were correlated with fewer hospitalizations and lower doses of radioactive iodine received, while basal TgAb and TSH had no relation. 

As is well established, periodic serum Tg measurement is the most sensitive test for the detection of recurrent or persistent disease during follow-up (1); however, TgAb interferes with this measurement (8). 

Previously reported percentages of TgAb in DTC patients range from 10% to 30% (6,7); however, the present study showed an increased frequency (34.9%) of thyroid autoantibody in these patients. The increased prevalence of thyroid antibodies in DTC might indicate an enhanced presence of thyroid tumour antigens to the immune system, although other studies report that tumour Tg has decreased antigenicity, resulting from lower iodine content (15). It is uncertain whether the thyroid antibodies at the time of DTC detection and initial surgery have any clinical implication (16). A small number of reports have reported that, among TgAb-positive patients, about 20%-30% have established recurrent DTC, suggesting that TgAb could indicate the presence of recurrent disease in these patients (5,6). 

Conversely, an immune response directed against thyroid cancer might be important in preventing metastasis and recurrence of diseases (17). In this field, previous observations have shown that adults with autoimmune thyroiditis or lymphocytic infiltration surrounding papillary thyroid carcinoma (PTC) have improved disease-free survival; therefore, some authors considerate TgAb as a good prognostic factor in DTC patients (17). 

Various studies in the literature have reported mixed results regarding the effect of TgAb in DTC patients’ outcomes (5,11,17). 

One study reports a correlation among lymphocytic infiltration, serum thyroid autoantibodies, and a favourable long-term outcome (18). 

In that study, in 95 patients with PTC, recurrence of the tumour was assessed according to lymphocytic infiltration in the thyroid gland (18). The researchers divided the patients into two groups: Group A consisted of 36 patients with PTC associated with lymphocytic infiltration, and group B consisted of the remaining 59 patients, with PTC with no lymphocytic infiltration (18). Antithyroglobulin antibody and/or antimicrosomal antibody were positive in 16 patients from group A and 4 patients from group B (p<0.001). Recurrence of the tumour was found in only one patient in group A (2.8%), but in 11 patients in group B (18.6%). The percentage of patients free from recurrence over a 10-yr follow-up in group A was significantly higher than that in group B (by Cox-Mantel test, p<0.01). They concluded that lymphocytic infiltration surrounding the tumour or inside the tumour in PTC might be of use as a mean for predicting favourable prognosis; patients with no lymphocytic infiltration had a high rate of recurrence (18). In another study, in 39 childhood PTC cases, 9 follicular thyroid carcinomas, 2 medullary thyroid carcinomas, 11 benign thyroid lesions, and 2 normal thyroid glands were assessed for the presence of lymphocytes and lymphocyte proliferation (17). The researchers concluded that proliferation of tumour-associated lymphocytes was associated with improved disease-free survival in children and young adults with thyroid cancer (17). The next detailed study demonstrated that the immune response against PTC was important and complex, involving more than one type of lymphocyte (19). 

On the other hand, some studies have shown that elevated TgAb levels appear to serve as a useful marker for recurrent or persistent DTC in patients with undetectable serum Tg results (5). 

The clinical significance of TgAb was assessed in 226 DTC patients who had undergone remnant ablation, and who displayed undetectable Tg levels (5). The researchers showed that 51 (22.6%) of the Tg undetectable patients showed positive TgAb, and 25 (49.0%) of these were confirmed with recurrence. The recurrence rate of TgAb-positive patients was higher than that of TgAb-negative patients (3.4%; p<0.0001). During follow-up, 73.1% of the disease-free patients showed spontaneously decreased TgAb levels (5). 

In another study, in 824 Tg negative patients with DTC, it was noted that serum TgAb levels measured at 6-12 months after remnant ablation could forecast recurrence in patients with undetectable Tg values (10). The researchers concluded that, in patients with undetectable Tg and with positive TgAb values, a change in TgAb concentration during the early postoperative period might be a prognostic factor of recurrence (10). 

A study by Spencer et al. (7), in accordance with other reports, showed that the presence of TgAb positivity during long term follow-up points out persistent disease, whereas the loss of TgAb positivity implies a surgical cure (8,11). 

As mentioned in the above studies, indicating TgAb level as a marker for recurrent or persistent disease in DTC patients was implemented only in patients with undetectable serum Tg results, but not in all groups, as in the current study. This point may necessary to consider in more well-designed investigations. 

Furthermore, our data indicate that basal serum Tg level correlated well with patient outcome and had a complementary role in predicting persistence or recurrence of disease in the earliest postoperative period. 

A number of investigations have addressed this issue. One study, which included 100 patients, was designed to check the value of post-operative and post-ablative serum thyroglobulin levels and diagnostic whole-body scan in predicting remission in DTC patients. The researchers demonstrated that post-operative and post-ablative serum TG levels (but not follow-up diagnostic WBS) had prognostic value and enabled the identification of patients with a higher risk of persistent/recurrent disease (20). 

Recently, a study of 170 DTC patients revealed that the post-ablation I-131 scintigraphy with neck and thorax SPECT-CT and stimulated serum Tg level enabled an early appraisal of the risk of persistent or recurrent disease in DTC patients (21). Malandrino et al. assessed this issue in 425 DTC patients and concluded that basal Tg allows for the identification of DTC patients who are likely to remain disease-free, with great accuracy. This simple measurement, therefore, may be sufficient to assess the risk-adapted management of DTC patients (22). 

A similar finding was found in a study of 268 low risk patients with DTC, treated with total or near-total thyroidectomy, followed by immediate I-131 remnant ablation (2). In this study, patients with anti-Tg autoantibodies, and those showing evidence of extra-cervical metastases, were excluded (2). These data indicated that serum Tg levels measured at the time of immediate postoperative I-131 remnant ablation correlated well with serum Tg levels at the time of the initial diagnostic WBS and had a complementary role in predicting persistence or recurrence of disease in the earliest postoperative period (2). 

As compared with our study, we included all the patients with TgAb, as well as high risk cases. 

Likewise, it seems that the Tg levels in the DTC patients are correlated with disease volume, histopathology of cancer, and metastatic locations. In a retrospective study of 417 DTC cases, the basal Tg level was correlated with disease volume, metastatic location (more in the bone metastases than lung or neck metastases) and cancer histology (more in follicular rather than papillary subtypes). In our study, we found the same results regarding basal Tg levels while TgAb levels were not different in the above subgroup (23). It could be indicated that TgAb level is unrelated to metastases or type of DTC. 

It should be mentioned that our study had some drawbacks. One of the most important limitations of this study is its small sample size. Another limitation is that we did not measure all autoimmune antibodies, such as anti-TPO, which may have interfered with our results. However, these findings support the claim that preablative Tg level and postablation I-131 scintigraphy, but not TgAb level, could be useful indicators of patient outcome, although further evidence needs to be obtained. 

## CONCLUSION

This study showed that basal serum Tg levels and post-ablation I-131 scintigraphy in patients with differentiated thyroid cancer were correlated well with patient outcomes. While the basal TgAb and TSH levels did not demonstrate such a relation, it seems that basal Tg levels could assist in classification of risk-adapted patients. 

## ACKNOWLEDGEMENTS

This study was the postgraduate thesis of Dr Ali Shafiepour Siahkali and was supported by sponsorship of Shahid Beheshti University of Medical Sciences (grant no. 4876).

## Figures and Tables

**Table 1:**
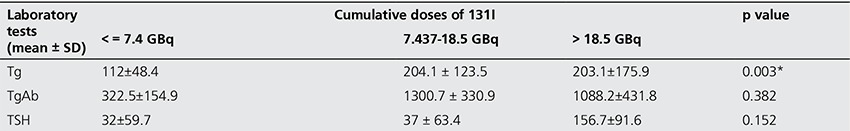
Comparison of basal Tg, TgAb and TSH levels in three different groups according to cumulative doses of 131I

**Table 2:**
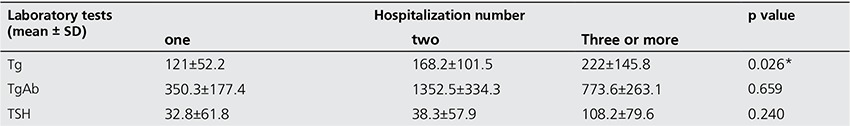
Comparison of basal Tg, TgAb and TSH levels in three different groups according to hospitalization number
